# Posterior Wall Isolation for Atrial Fibrillation: Excessive or Necessary?

**DOI:** 10.19102/icrm.2018.090606

**Published:** 2018-06-15

**Authors:** Rahul N. Doshi

**Affiliations:** ^1^Division of Cardiology, Keck USC School of Medicine, Los Angeles, CA, USA

**Keywords:** Ablation, atrial fibrillation, posterior wall

Moderation is a fatal thing. Nothing succeeds like excess (Oscar Wilde).

Everything in excess is opposed to nature (Hippocrates).

In this month’s issue of *The Journal of Innovations in Cardiac Rhythm Management*, Tahir et al. provide a concise yet comprehensive review of the rationale and evidence supporting complete left atrial posterior wall isolation (PWI) as an ablative strategy in the treatment of atrial fibrillation (AF).^1^

The rationale behind PWI is compelling, and the authors provide a laundry list of explanations as to why this technique makes sense. Considering its embryologic origin, ganglionated plexi, electrophysiologic properties, fibrosis, and anisotropy, the posterior wall seems like it was designed to promote AF. The clinical basis is compelling as well. The high success rates seen with the surgical Cox maze procedure included the performance of PWI as well.^2^ Clinical data in humans demonstrate both dominant frequencies and rotors localizing most commonly on the posterior wall.^3,4^ Single-center trials, especially with regard to treating persistent AF, have consistently shown the ability to isolate the posterior wall and demonstrate higher success rates for AF-free survival.^5,6^ Indeed, many centers (including ours) routinely isolate the posterior wall in patients with persistent AF. The application of PWI in patients with paroxysmal AF is more variable. Our approach is to perform the procedure in patients with extensive fibrosis (or a surrogate such as low voltage) or during redo procedures.

## Trouble in paradise

So, where does the controversy lie? Tahir et al. summarized the available clinical studies on this topic quite well.^1^ PWI does require a greater number of lesions and more time. Though an increase in the number of lesions could elevate the risk of collateral damage in general, the controversy lies in the location of this increased ablation strategy—the posterior wall. Importantly, with posterior wall ablation comes the possibility of esophageal injury.

Atrioesophageal fistula is the Keyser Söze of atrial fibrillation ablationists (forgive the reference). We know it exists, rare as it may be. We know what causes it—sort of. The difficulty lies in how to prevent it. We limit power and contact force. We monitor esophageal luminal temperature. We prophylactically treat with antacid therapy. Unfortunately, despite these efforts, there remains a gnawing feeling of uncertainty, which can adversely affect our ability to embrace a potential therapy.

This devastating complication is associated with complete uncertainty. The studies referenced by Tahir et al.^1^ do not suggest any increased risk of fistula formation. However, given the rare nature of this entity, we cannot assess whether or not our strategies reduce or eliminate the risk. The authors mentioned the limitations of esophageal temperature monitoring. A meta-analysis performed by Koranne et al. failed to demonstrate any reduction in the incidence of atrioesophageal fistula with esophageal temperature monitoring.^7^ There is even uncertainty when it comes to making the diagnosis. In a recent study, Ha et al. performed an extensive literature review of reported cases and found that the only method of certainty is the presence of blood culture positivity, particularly with respect to streptococcal species.^8^ Computed tomography imaging was successfully diagnostic in only one-third of cases.

Perhaps we need to accept that all catheter ablation procedures are associated with some degree of uncertainty and not be afraid of the excess. Without direct tissue imaging in real time, we are left with only surrogates of ablation success approximating lesion depth or transmurality. Temperature, power, impedance drops, and force–time intervals all have their inherent limitations. However, we can take solace in clinical endpoints. Take force-sensing catheters as an example. There is a concern that these catheters are associated with a higher risk of collateral damage by way of creating deeper lesions.^9^ However, Lin et al. performed a recent meta-analysis, demonstrating that this technology facilitates pulmonary vein isolation and results in more favorable outcomes and/or higher rates of AF-free survival without any increase in procedural-related complications.^10^ In fact, the overall complication rate for AF ablation appears to be decreasing in comparison with that seen a decade ago.^11^

When in doubt, we turn to data. In their manuscript, Tahir et al.^1^ referred to two small, randomized clinical trials, one showing a benefit and one not, that are fueling the controversy.^12,13^ The authors pointed out the heterogeneous population in the latter study and suggested that the benefit of this approach might be driven by more rigorous patient selection. Approaches such as late gadolinium enhancement on cardiac magnetic resonance imaging can identify substrates and certainly have great promise in the selection of suitable patients and in validating the potential need for posterior wall ablation.^14^ However, data suggest the need for a large multicenter prospective randomized clinical trial that perhaps uses some criteria for patient selection beyond AF type or burden.

In the right patient, the creation of additional lesions may be the best solution to increase efficacy and decrease the need for repeat procedures. However, we clearly need more clinical evidence that our treatment strategies confer benefit without significantly increasing risk. In the meantime, we must continue to use our own individual judgments, complete with all of their inherent biases and accompanying sense of anxiety, in order to provide patients with the best care possible at this stage.

My final quote: *sometimes too much to drink is barely enough (Mark Twain).*

## References

[1] Tahir KS, Mounsey JP, Hummel JP (2018). Posterior wall isolation in atrial fibrillation ablation. J Innov Cardiac Rhythm Manage.

[2] Cox JL, Ad N, Palazzo T (2000). Current status of the maze procedure for the treatment of atrial fibrillation. Semin Thorac Cardiovasc Surg.

[3] Mandapati R, Skanes A, Chen J, Berenfeld O, Jalife J (2000). Stable microreentrant sources as a mechanism of atrial fibrillation in the isolated sheep heart. Circulation.

[4] Narayan SM, Krummen DE, Clopton P (2013). Direct or coincidental elimination of stable rotors or focal sources may explain successful atrial fibrillation ablation: on-treatment analysis of the CONFIRM trial (Conventional Ablation for AF With or Without Focal Impulse and Rotor Modulation). J Am Coll Cardiol.

[5] Sanders P, Hocini M, Jais P (2007). Complete isolation of the pulmonary veins and posterior left atrium in chronic atrial fibrillation. Long-term clinical outcome. Eur Heart J.

[6] Bai R, DiBiase L, Mohanty P (2016). Proven isolation of the pulmonary vein antrum with or without left atrial posterior wall isolation in patients with persistent atrial fibrillation. Heart Rhythm.

[7] Koranne K, Basu-Ray I, Parikh V (2016). Esophageal temperature monitoring during radiofrequency ablation of atrial fibrillation: a meta-analysis. J Atr Fibrillation.

[8] Ha FJ, Han HC, Sanders P (2018). Challenges and limitations in the diagnosis of atrio-esophageal fistula. J Cardiovasc Electrophysiol.

[9] Black-Maier E, Pokorney SD, Barnett AS (2017). Risk of atrioesophageal fistula formation with contact force–sensing catheters. Heart Rhythm.

[10] Lin H, Chen YH, Hou JW (2017). Role of contact force-guided radiofrequency catheter ablation for treatment of atrial fibrillation: a systematic review and meta-analysis. J Cardiovasc Electrophysiol.

[11] Muthalaly RG, John RM, Schaeffer B (2018). Temporal trends in safety and complication rates of catheter ablation for atrial fibrillation. J Cardiovasc Electrophysiol.

[12] Kim J, Shin SY, Na Jo (2015). Does isolation of the left atrial posterior wall improve clinical outcomes after radiofrequency catheter ablation for persistent atrial fibrillation? A prospective randomized clinical trial. Int J Cardiol.

[13] Tomborero D, Mont L, Berruezo A (2009). Left atrial posterior wall isolation does not improve the outcome of circumferential pulmonary vein ablation for atrial fibrillation: a prospective randomized study. Circ Arrhythm Electrophysiol.

[14] Higuchi K, Cates J, Gardner G (2018). The spatial distribution of late gadolinium enhancement of left atrial magnetic resonance imaging in patients with atrial fibrillation. JACC Clin Electrophysiol.

